# G6PC3 is involved in spermatogenesis by maintaining meiotic sex chromosome inactivation: Meiotic functions of G6PC3

**DOI:** 10.3724/abbs.2024172

**Published:** 2024-10-16

**Authors:** Yuming Cao, Shengnan Wang, Liyang Li, Wenwen Li, Yan Liang, Fei Ao, Zexiao Wei, Li Wang

**Affiliations:** Department of Obstetrics and Gynecology Perinatal Medical Center the Fifth Affiliated Hospital of Sun Yat-sen University Zhuhai 519000 China

**Keywords:** glucose 6 phosphatase catalytic 3 (G6PC3), spermatogenesis, sex chromosomes, pachytene arrest, meiotic sex chromosome inactivation

## Abstract

Meiosis, a process unique to germ cells, involves formation and repair of double-stranded nicks in DNA, pairing and segregation of homologous chromosomes, which ultimately achieves recombination of homologous chromosomes. Genetic abnormalities resulted from defects in meiosis are leading causes of infertility in humans. Meiotic sex chromosome inactivation (MSCI) plays a crucial role in the development of male germ cells in mammals, yet its underlying mechanisms remain poorly understood. In this study, we illustrate the predominant presence of a protein known as glucose 6 phosphatase catalyzed 3 (G6PC3) in pachytene spermatocytes, with a high concentration in the sex body (XY body), suggesting its significant involvement in male germ cell development. By employing CRISPR-Cas9 technology, we generate mice deficient in the *G6pc3* gene, resulting in complete meiotic arrest at the pachytene stage in spermatocytes and are completely sterile. Additionally, we observe abnormal XY body formation and impaired MSCI in *G6pc3-*knockout spermatocytes. These findings underscore *G6pc3* as a new essential regulator that is essential for meiotic progression. G6PC3 is involved in spermatocyte during male spermatogenesis development by the maintenance of meiosis chromosome silencing.

## Introduction

To create haploid gametes, both paternal and maternal germ cells undergo a process called meiosis, which is characterized by the pairing and exchange of genetic material between their corresponding chromosomes [1]. The accuracy of meiosis is tightly regulated by checkpoint mechanisms, ensuring that cells with faulty chromosome pairing are eliminated to prevent the production of gametes with an incorrect number of chromosomes [2]. In male meiosis, sex chromosomes form a specialized structure known as the sex body (XY body), where they undergo widespread transcriptional silencing, a phenomenon referred to as meiotic sex chromosome inactivation (MSCI) [3]. MSCI is a critical event in male germ cell development because of the evolution of distinct sex chromosomes in mammals [4].

Many important components involved in MSCI have been identified, including sensing proteins such as SYCP3, HORMAD1, HORMAD2, and BRCA1, as well as effector molecules such as MDC1 and histone γH2AX [ 5– 8]. Despite the well-established link between impaired MSCI and meiotic defects leading to germ cell elimination, the specific mechanisms underlying this process remain poorly understood.

Glucose 6 phosphatase catalyzed 3 (G6PC3) is a significant membrane protein found in the endoplasmic reticulum that consists of 9 transmembrane regions [9]. The active site of the catalytic subunit of glucose-6-phosphatase faces the interior of the endoplasmic reticulum, while the glucose-6 phosphatase (G6P) transporter facilitates the transport of G6P molecules from the cytoplasm to the catalytic subunit’s active site [10]. G6PC3 is implicated in various physiological processes, such as cardiovascular function, urogenital health, neutropenia, and even testicular failure [11]. G6PC3 is highly conserved across various species [12], However, its role in reproductive development has not been fully elucidated.

In this study, we utilized *G6pc3* ^+ / + ^ and *G6pc3* ^‒/‒^ mice to systematically investigate the crucial role of *G6pc3* in mouse spermatogenesis. We observed that *G6pc3* is specifically and predominantly expressed in mouse testicular tissue; however, its significance in spermatogenesis remains unexplored. Consequently, we generated *G6pc3*-knockout mice with targeted gene disruption, revealing that the sperms from *G6pc3*-knockout mice are arrested at the spermatocyte stage due to defects in meiotic chromosome silencing.

## Materials and Methods

### Mice

*G6pc3* ^+ /‒^ mice were constructed by Saiye Biotechnology ( https://www.cyagen.com/cn/zh-cn/about-us.html; Suzhou, China), and heterozygous mice were bred at the Fifth Affiliated Hospital of Sun Yat-sen University. Using the CRISPR-Cas9 strategy, we generated *G6pc3*-deleted mice on a C57BL/6 genetic background by targeting exons 1–6 with two sgRNAs, resulting in a frameshift mutation of 4100 bp and an early stop codon. All the mice were maintained under specific pathogen-free (SPF) conditions with a 12-h/12-h light-dark cycle. Each experimental parameter was replicated at least three times. Animal experiments were conducted in accordance with the guidelines of the Animal Ethics Committee of the Fifth Affiliated Hospital of Sun Yat-sen University (Approval number: 00341). Genotyping was carried out via PCR amplification of genomic DNA extracted from mouse tails, with the *G6pc3* mutant allele detected via specific primers: F1: 5′-CCCTGGAGATTAGACATCGACTT-3′; F2: 5′-GATCAGCCTCTTCTACATCTTCAAC-3′; R1: 5′-AAGAATTCACAAGTTCCAAGGGC-3′.

### Litter size and cauda epididymis sperm count

To assess litter size and cauda epididymis sperm count, wild-type, heterozygous, and *G6pc3*-knockout mice were housed in cages containing 2 females and 1 male, with 3 cages per group. The litter size of the 9 cages was recorded, and the litter size for each genotype was calculated over 6 months of continuous observation. Male mice with the *G6pc3* ^+ / + ^ or *G6pc3* ^‒/‒^ genotype were euthanized at 8 weeks of age via cervical dislocation. The testicular tissue and the epididymal tail were surgically removed. Testicles were cleaned and weighed individually, and the epididymal tail was placed in 1 mL of PBS (G4202; Servicebio, Wuhan, China). The epididymal tail was cut into small pieces to release the sperm fully, and 10 μL of the sperm suspension was used to count the sperm on a sperm counting board for both groups of mice, with five mice in each group.

### Western blot analysis

For western blot analysis, male mice with the *G6pc3* ^+ / + ^ or *G6pc3* ^‒/‒^ genotype were euthanized at 8 weeks of age via cervical dislocation. Testicular tissue was surgically collected and crushed on ice using Radio Immunoprecipitation Assay (RIPA) Lysis buffer (P0013B; Beyotime, Shanghai, China). A protease inhibitor cocktail (P1005; Beyotime) was added during protein isolation to prevent cleavage. The protein concentration was determined via a BCA protein concentration assay kit (P0009; Beyotime). SDS-PAGE (10%) was performed on the proteins, followed by protein transfer onto a PVDF membrane (IPVH00010; Merck Millipore, Billerica, USA). The membrane was subsequently incubated with primary antibodies against G6PC3 (A16234, 1:1000; Abclonal, Wuhan, China) and β-actin (AC026, 1:10000; Abclonal), followed by incubation with an HRP-conjugated secondary antibody (AS016, AS003, 1:8000; Abclonal). Finally, enhanced chemiluminescence (ECL) reagent (RM00021; Abclonal) was used for development.

### qRT-PCR

At 8 weeks of age, male *G6pc3* ^+ / + ^ mice were euthanized via cervical dislocation, and tissues, including liver, heart, spleen, lung, kidney, brain, testis, and epididymis, were surgically collected. Total RNA was extracted from these tissues using the RNA extraction kit (RK30120; Abclonal). To assess the expression level of *G6pc3* mRNA across different tissues, equal amounts of cDNA were synthesized via the PrimeScript RT reagent kit with Genomic DNA Eraser (R323-01; Vazyme, Nanjing, China). The target gene expression was determined by using SYBR Green qPCR Master Mix (Q221-01; Abclonal). *β-Actin* was used as the housekeeping gene. The 2 ^–ΔΔCt^ method was applied to determine the relative gene expression. The sequences of primers used for amplification were as follows: *C6pc3*-F: 5′-CTGCCCTTGGCTGGCTAAT-3′, *G6pc3*-R: 5′-ATTCCAGGAAGACCAGCAGC-3′; *β-actin*-F: 5′-CTTAGTTGCGTTACACCCTTTC-3′, *β-actin*-R: 5′-CACCTTCACCGTTCCAGTTT-3′.

### Histological analysis and TUNEL assay

After dissection, the testes were immersed in 4% paraformaldehyde (DF0135; Leagene, Beijing, China) overnight at 4°C for fixation. The samples were then dehydrated via sequential immersion in 70%, 80%, or 90% ethanol solutions for 30 min each, followed by immersion in 95% or 100% ethanol for 20 min each. The samples were subsequently placed in a mixture of xylene and paraffin for 15 min, followed by immersion in paraffin I and paraffin II for 60 min each. Tissue sections (4 μm thick) were prepared via a microtome. The paraffin-embedded sections were then deparaffinized and rehydrated via a descending series of ethanol. Finally, the sections were stained with hematoxylin and eosin (C0105S; Beyotime) for histological examination, and images were captured via a BX53 microscope (Olympus, Tokyo, Japan). To detect the apoptosis of germ cells in the testicular tissues of *G6pc3* ^+ / + ^ and *G6pc3* ^‒/‒^ mice, a TUNEL assay was performed on testicular sections using a cell death detection kit (11684795910; Roche, Basel, Switzerland) according to the manufacturer’s instructions. Immunofluorescence images were captured via an Axio Observer 3 microscope (Zeiss, Oberkochen, Germany).

### Immunofluorescence analysis

For surface nuclear spread analysis, spermatocytes from P21 testes were utilized following previously established procedures. Immunofluorescence staining was performed on spread nuclei or frozen sections using the following primary antibodies: mouse anti-G6PC3 (A16234, 1:100; ABclonal), rabbit anti-SYCP1 (ab15090, 1:200; Abcam, Cambridge, UK), mouse anti-SYCP3 (ab97672, 1:200; Abcam), mouse anti-γH2AX (05-636, 1:200; Millipore), RNA polymerase II (ab5131, 1:100; Abcam), rabbit-histone H1T (ab61177, 1:100; Abcam), mouse anti-DDX4 (ab27591, 1:100; Abcam) and rabbit anti-H3K4me3 (A22146, 1:100; Abcam). The sections were then incubated with Alexa Fluor 488- or 594-conjugated goat anti-rabbit (or mouse) IgG antibodies (ab150084, ab150077, ab150113, and ab150120, 1:400 dilution; Abcam) for 1 h at room temperature. The slides were subsequently washed three times with PBS and mounted with antifade mounting medium containing DAPI (ZLI-9557; ZSGB-BIO, Beijing, China). Immunofluorescence images were captured via an Axio Observer 3 microscope (Zeiss).

### RNA-Seq and data analysis

Total RNA was extracted from pachytene spermatocytes obtained from *G6pc3* ^+ / + ^ and *G6pc3* ^‒/‒^ mice via the STA-PUT method using the TRIzol reagent (Invitrogen, Carlsbad, USA). The concentration and integrity of the RNA were assessed via a Qubit 2.0 fluorometer (Invitrogen) and a Bioanalyzer 2100 system (Agilent, Santa Clara, USA). First-strand cDNA was synthesized via random hexamer primers and M-MuLV reverse transcriptase, followed by treatment with RNaseH to degrade the RNA. Subsequently, second-strand cDNA synthesis was performed via DNA polymerase I and dNTPs. PCR amplification was carried out via Phusion high-fidelity DNA polymerase, universal PCR primers, and Index (X) primers. The libraries were constructed via a kit (NEBNext® Ultra™ RNA Library Prep Kit; Illumina, San Diego, USA) according to the manufacturer’s protocol. Sequencing was performed on the Illumina HiSeq 4000 system (Illumina). The sequencing depth was 6G, and the read length was 100 bp. Gene expression was quantified via kallisto ( https://github.com/pachterlab/kallisto). Transcript abundance (counts) was summarized per gene via tximport ( https://bioconductor.org) and then imported for performing gene expression analysis via edgeR [13].

### Statistical analysis

The experiments were repeated at least three times. Statistical analysis was performed via GraphPad Prism 8.0.2 (GraphPad Software, La Jolla, USA). The statistical analysis results were presented using the Student’s *t*-test. Data are expressed as the mean ± SD. *P*  < 0.05 indicated significant statistical difference.

## Results

### G6PC3 is a male testis-specific protein that highly accumulates on the XY body of spermatocytes

**Figure 1:**
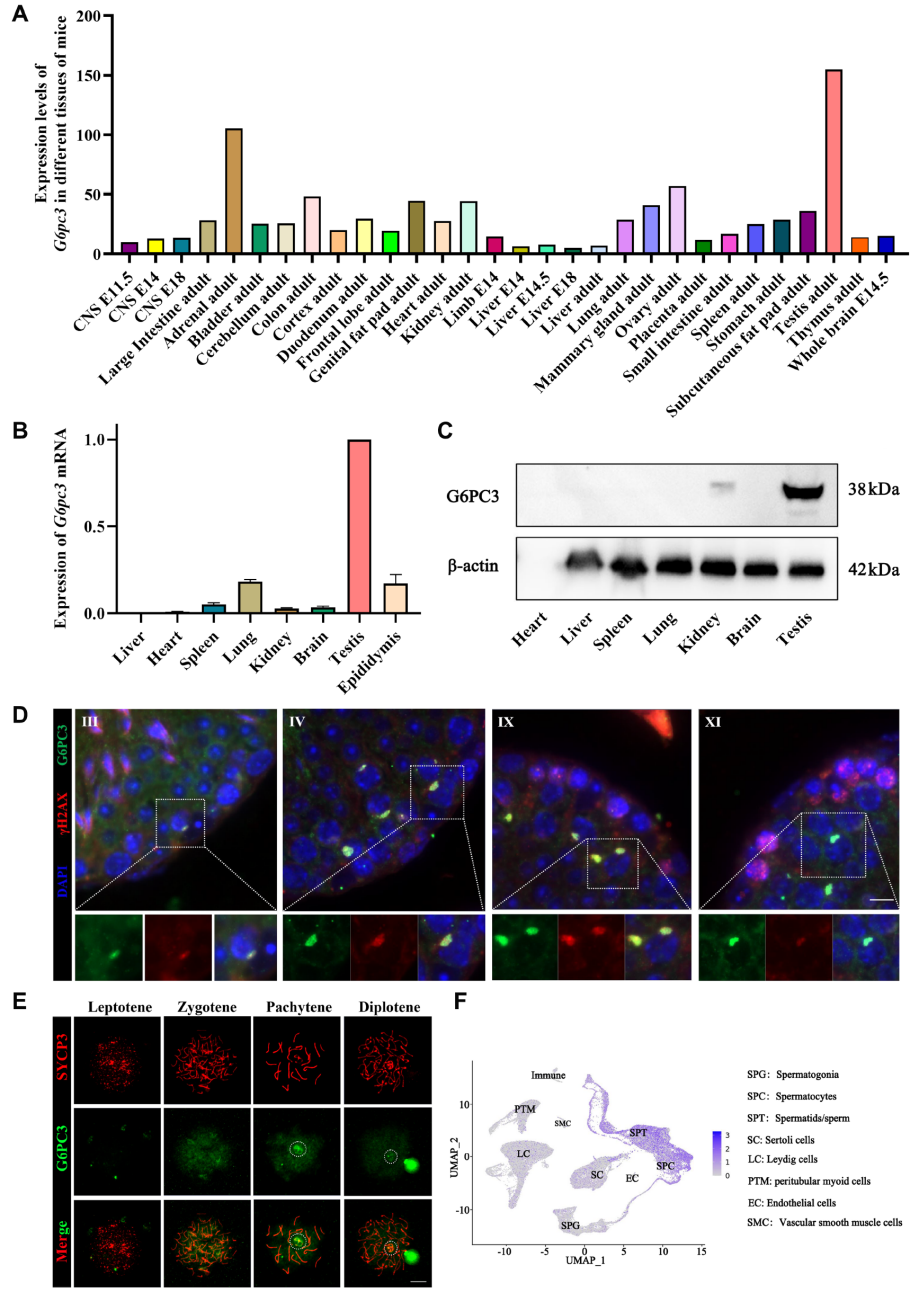
Figure 1 G6PC3 is a testis-specific protein that accumulates on the XY body in spermatocytes (A) NCBI database analysis of the expression profile of G6pc3 mRNA in mouse tissues. (B) qRT-PCR verification of G6pc3 mRNA levels in multiple mouse tissues. Gapdh was used as a housekeeping gene, and the results were processed via the 2–ΔΔCT method. Data are presented as the mean ± SD, n = 3. (C) G6PC3 expression profile in adult (P56) mouse tissues detected by western blot analysis. β-Actin served as a loading control. (D) Immunofluorescence staining analysis of G6PC3 in testis sections. γH2AX was used as a marker for spermatocytes. The nuclei were stained with DAPI. The inset is an enlarged view of a pachytene spermatocyte with the XY body indicated. Scale bar: 50 μm. (E) Immunostaining of G6PC3 (red) and SYCP3 (green) on chromosome spreads of spermatocytes from P21 G6pc3 + / +  testes; the white arrow indicates the XY body, n = 3 mice for each group. Scale bar: 10 μm. (F) The expression pattern of G6pc3 in the mouse germline atlas was analyzed via a single-cell sequencing database (http://malehealthatlas.cn/).

To explore the biological function of G6PC3, we analyzed the expression and localization of G6PC3 during germ cell development. The NCBI database revealed high specific expression of G6PC3 in mouse testicular tissue ( Figure 1A). Both qRT-PCR and western blot analyses further confirmed that the G6PC3 protein is predominantly expressed in the testes of male mice (P56), with minimal expression detected in other tissues ( Figure 1B,C). Immunofluorescence analysis of testicular slices from adult male mice revealed that G6PC3 initially exhibited diffuse expression in pachytene stage spermatocytes and showed limited accumulation on XY bodies labeled with the γH2AX signal ( Figure 1D). Furthermore, the accumulation of G6PC3 on the XY body rapidly diminished in diplotene spermatocytes ( Figure 1E). The results from the Male Health Atlas (MHA) single-cell database also revealed the specific expression of G6PC3 in spermatocytes ( Figure 1F). These findings suggest the potential involvement of G6PC3 in spermatocyte development or XY body formation.

### Global knockout of *G6pc3* in mice results in meiotic arrest at the pachytene stage

**Figure 2:**
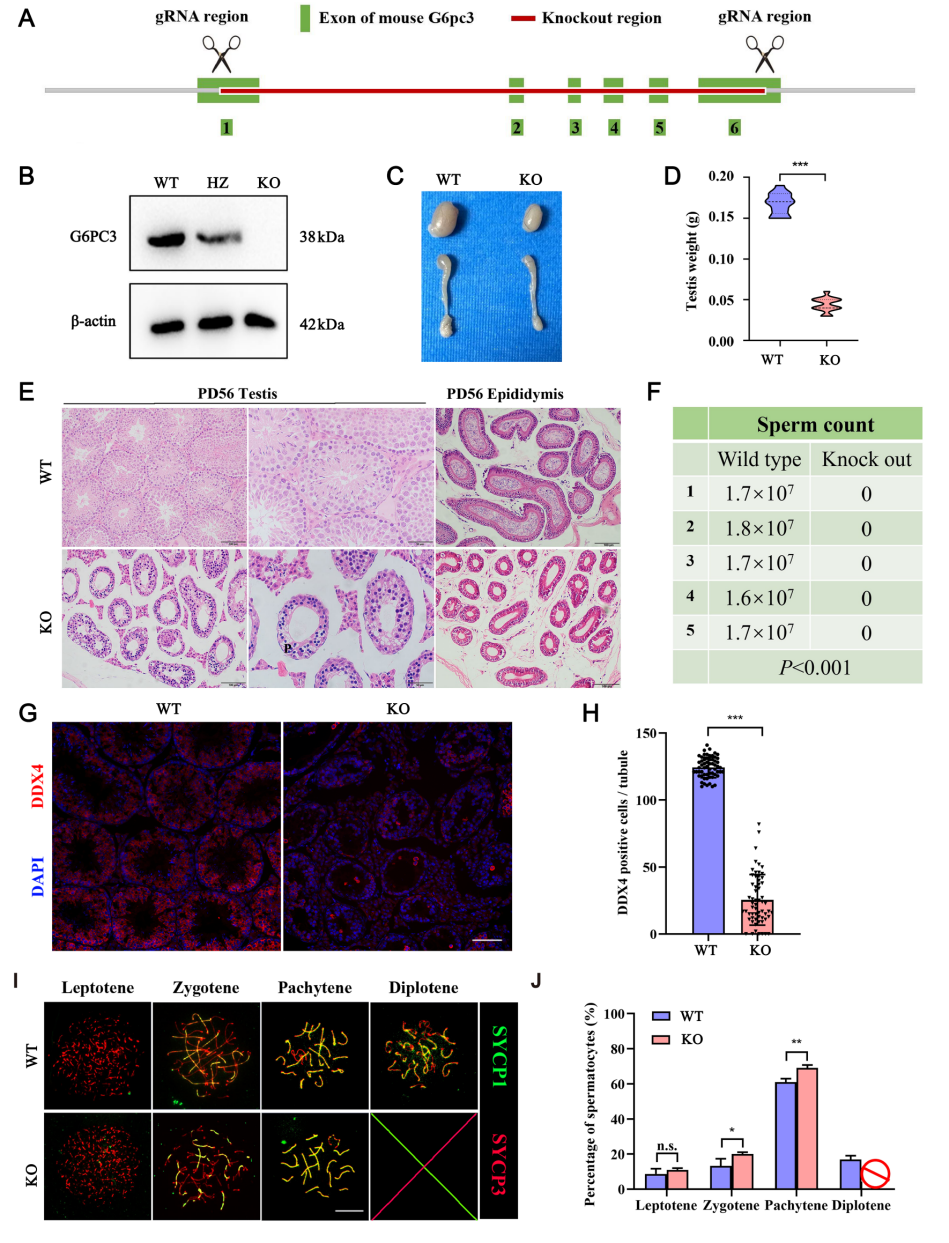
Figure 2 G6PC3 is essential for spermatocyte development (A) Schematic diagram of the generation of G6pc3-deficient mice with the CRISPR-Cas9 genome editing system. (B) Western blot analysis of G6PC3 protein expression in testis extracts from G6pc3 + / +  and G6pc3‒/‒ testes at P56. β-Actin served as a loading control. (C,D) Litter size (right) and weight (left) of G6pc3 + / +  and G6PC3‒/‒ testes at P56. n = 13. (E) Hematoxylin and eosin (H&E) staining of histological sections of testes from G6pc3 + / +  and G6pc3‒/‒ mice at P56. Scale bar, left: 100 μm, right: 50 μm. (F) Sperm count in the tails of the epididymides of G6pc3 + / +  and G6pc3‒/‒ mice. P < 0.001, n = 5. (G) Frozen section staining of G6PC3 and DDX4 on the meiotic chromosomes in G6pc3 + / +  and G6pc3‒/‒ testicular tissue. Scale bar: 50 μm. (H) Statistical analysis of the number of seminiferous tubules without DDX4. Data are presented as the average percentage; n = 3 mice for each group, and 100 tubules were counted for each mouse. (G) Immunostaining of SYCP1 (green) and SYCP3 (red) on chromosome spreads of spermatocytes from P35 G6pc3 + / +  and G6pc3‒/‒ testes. n = 3 mice per group. Scale bar: 10 μm. (H) Frequency of meiotic prophase I stages. n = 3 mice per group, and 50 spermatocytes from each mouse were examined. Data are presented as the mean ± SD, *P < 0.05, **P < 0.01, ***P < 0.001 by two-tailed Student’s t test. WT, wild type; KO, knockout; HZ, heterozygote.

To investigate the biological function of G6PC3 in spermatocyte development, we implemented a CRISPR-Cas9-mediated genome editing approach to disrupt *G6pc3* ( *G6pc3* ^‒/‒^) in mice ( Figure 2A). Western blot analysis confirmed the absence of the G6PC3 protein in *G6pc3* ^‒/‒^ testes ( Figure 2B). Notably, the physical development of *G6pc3* ^‒/‒^ mice was similar to that of their *G6pc3* ^+ / + ^ counterparts. However, at P56, the testes of *G6pc3* ^‒/‒^ mice were significantly smaller than those of their wild-type control littermates ( *P*  < 0.001, Figure 2C,D). Histological examination revealed that the seminiferous tubules of *G6pc3* ^‒/‒^ mice lacked meiotic spermatocytes at P56 ( Figure 2E), and the epididymal tail lacked sperm ( Figure 2F). Immunofluorescence staining for DDX4 (also known as MVH, a germ cell-specific marker) in frozen testis sections revealed a significant decrease in the number of DDX4-positive cells in *G6pc3* ^‒/‒^ testes compared with those in *G6pc3* ^+ / + ^ testes ( *P*  < 0.001, Figure 2G,H), indicating a loss of germ cells in *G6pc3* ^‒/‒^ testes. Analysis of chromosome synapse transverse elements (SCs) indicating SYCP1, synaptic complexes, and axial elements of SYCP3 and SC through nuclear diffusion revealed that *G6pc3* ^‒/‒^ spermatocytes progressed only to the pachytene phase and did not proceed to the diploid phase ( *P*  < 0.05, Figure 2I,J). These findings underscore the essential role of G6PC3 in the development of pachytene spermatocytes.

### G6PC3 is essential for the formation of XY bodies

**Figure 3:**
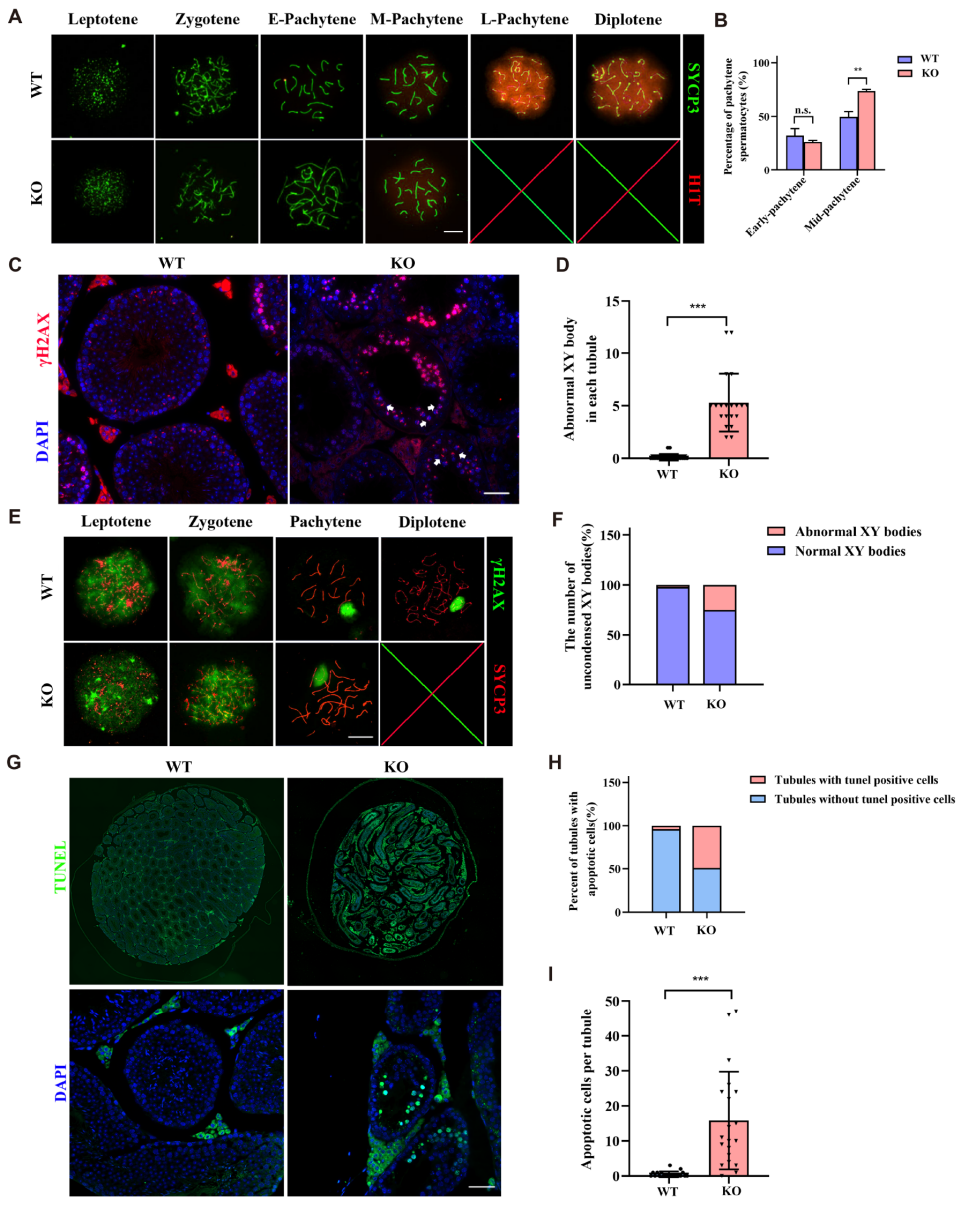
Figure 3 G6PC3 is essential for the formation of XY bodies (A) Immunostaining of SYCP3 (red) and H1T (green) on chromosome spreads of spermatocytes from P35 G6pc3 + / +  and G6PC3‒/‒ testes. n = 3 mice for each group. Scale bar: 10 μm. (B) Statistical analysis of the percentage of H1T-positive pachytene spermatocytes at different stages. Data are presented as the average percentage; n = 3 mice per group, and 100 pachytene spermatocytes were counted per mouse. (C) Immunostaining of γH2AX and DAPI in P56 G6pc3 + / +  and G6pc3‒/‒ testis frozen sections. Scale bar: 50 μm. (D) The number of abnormal XY bodies in each tubule of P56 G6pc3 + / +  and G6pc3‒/‒ mice. n = 3 mice for each group, and 20 tubules from each mouse were analyzed. (E) Immunostaining of SYCP3 (red) and γH2AX (green) in G6pc3 + / +  and G6pc3‒/‒ pachytene spermatocytes. Arrowhead: example of an extended XY pair. Scale bar, 10 μm. (F) Statistical results of (E). Data are presented as the average percentage; n = 3 mice for each group, and 50 pachytene spermatocytes were counted for each mouse. (G) TUNEL assays of testes sections prepared from 3-week-old G6pc3 + / +  and G6pc3‒/‒ mice. Scale bar, above: 100 μm, under: 50 μm. (H) Quantification of the number of TUNEL-positive seminiferous tubules. n = 3 mice for each group, and 50 spermatocytes were counted for each mouse. (I) Quantification of the number of TUNEL-positive cells per tubule. Twenty tubules per mouse were counted, and three mice from each genotype were analyzed. Data are presented as the mean ± SD. **P < 0.001, ***P < 0.001 by two-tailed Student’s t test.

To further delineate the stage of spermatocyte arrest in the pachytene phase in *G6pc3* ^‒/‒^ mice, we employed an H1T antibody for immunofluorescence staining. H1T is known to be expressed in cells during the middle and late stages of the pachytene phase, with significantly increased expression observed in the late stages. Our findings revealed that *G6pc3* ^‒/‒^ spermatocytes could progress to the mid pachytene stage but were unable to advance to the late pachytene stage ( *P*  < 0.01, Figure 3A,B). Immunofluorescence staining of γH2AX, a marker for incompletely synapsed X-Y chromosomes with unrepaired double-strand breaks (DSBs) at the pachytene stage in testicular sections, revealed that some *G6pc3* ^‒/‒^ pachytene spermatocytes presented fragmented or expanded XY bodies ( *P*  < 0.001, Figure 3C,D). Further staining of spread nuclei from *G6pc3* ^*‒*/ *‒*^ pachytene spermatocytes confirmed the diffuse localization pattern of γH2AX signals in approximately 25% of the observed pachytene spermatocytes, indicating abnormal condensation of the XY chromosomes ( *P*  < 0.001, Figure 3E,F). A terminal deoxynucleotidyl transferase dUTP nick end labeling (TUNEL) assay revealed a sharp increase in the number of apoptotic cells in the tubules of *G6pc3* ^‒/‒^ testes ( Figure 3G‒I), suggesting the elimination of spermatocytes in the mutants via apoptosis.

### Abnormal XY bodies undergo MSCI defects in *G6pc3* ^‒/‒^ mice

**Figure 4:**
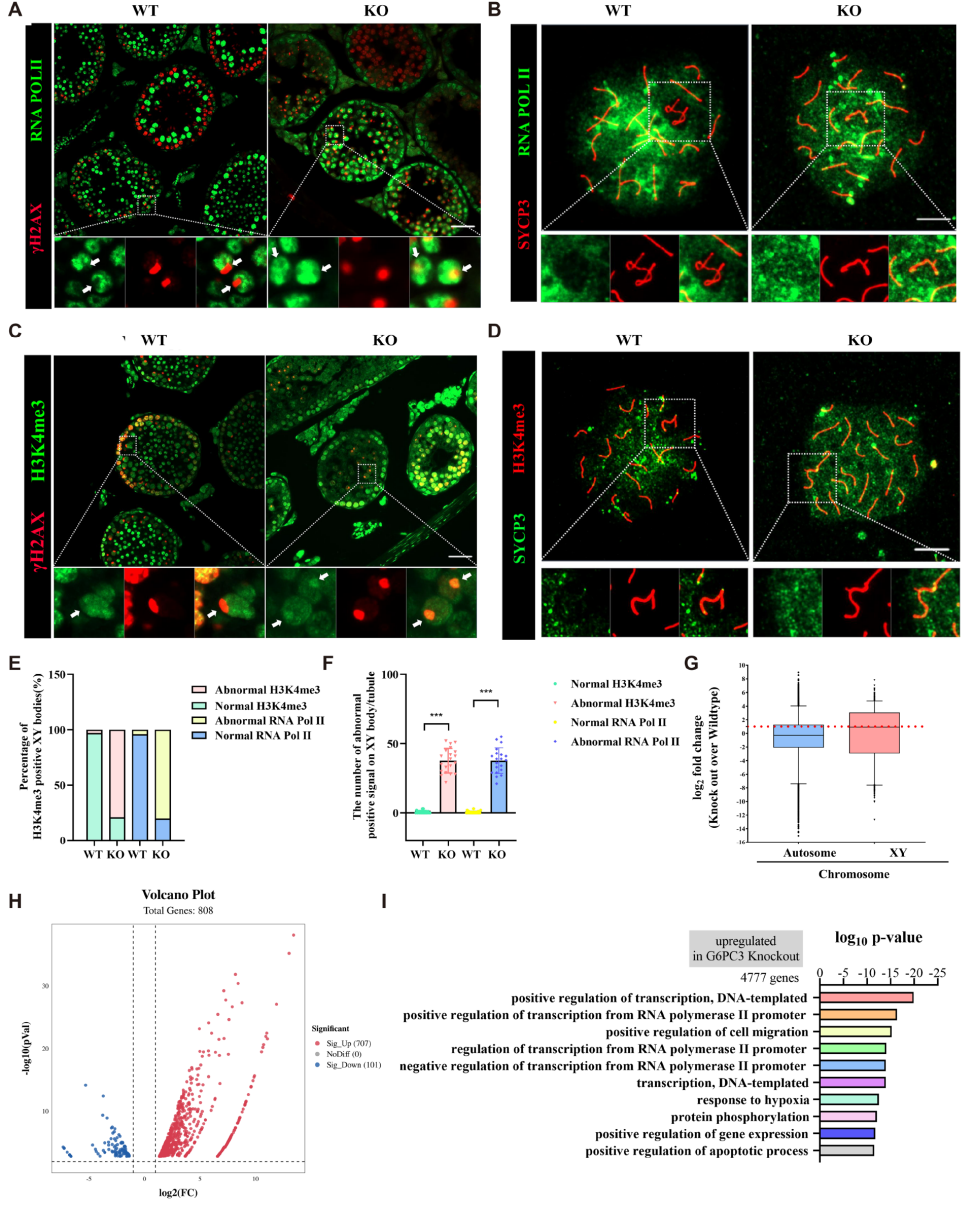
Figure 4 Abnormal XY bodies undergo MSCI defects in *G6pc3* ^‒/‒^ mice (A) Immunostaining of meiotic sex chromosome silencing in G6pc3 + / +  and G6pc3‒/‒ testis sections (γ-H2AX: red; RNA-Pol II: green). Scale bar: 50 μm. (B) Immunostaining of meiotic sex chromosome silencing in G6pc3 + / +  and G6pc3‒/‒ spermatocytes at the pachytene stage (SYCP3: red; RNA Pol II: green). Scale bar: 10 μm. (C) Immunostaining of H3K4me3 revealed active transcription of the XY chromosomes in G6pc3 + / +  and G6pc3‒/‒ testis sections (γ-H2AX: red; H3K4me3: green). Scale bar: 50 μm. (D) Immunostaining of H3K4me3 revealed active transcription of the XY chromosomes in G6pc3 + / +  and G6pc3‒/‒ spermatocytes at the pachytene stage (SYCP3: red; H3K4me3: green). Scale bar: 10 μm. (E) Statistical results of A and C. Data are presented as the average percentage; n = 3 mice for each group, and 50 pachytene spermatocytes were counted for each mouse. (F) Statistical results of B and D. Data are presented as the average percentage; n = 3 mice for each group, and 50 pachytene spermatocytes were counted for each mouse. (G–H) RNA-seq analysis of differential gene expression in testicular tissues of G6pc3 + / +  and G6pc3‒/‒ mice. (I) Functional annotation of DEGs between G6pc3 + / +  and G6pc3‒/‒ spermatocytes on the basis of RNA-seq data. ***P < 0.001.

The presence of abnormal XY bodies in *G6pc3* ^*‒*/ *‒*^ pachytene spermatocytes prompted us to investigate whether meiotic sex chromosome inactivation (MSCI) is affected by *G6pc3* deletion. We conducted RNA polymerase II staining on both frozen testicular sections and spread nuclei from pachytene spermatocytes. As anticipated, we observed that the RNA polymerase II signal persisted on the XY body in *G6pc3* ^*‒*/ *‒*^ pachytene spermatocytes, whereas RNA polymerase II was excluded from the XY body in *G6pc3* ^+ / + ^ pachytene spermatocytes ( Figure 4A,E). The number of RNA polymerase II-positive signals on the XY body within single seminiferous tubules in *G6pc3* ^+ / + ^ mice was significantly greater than that in *G6pc3* ^*‒*/ *‒*^ mice ( *P*  < 0.001, Figure 4B,F). Additionally, we performed immunofluorescence staining of the histone modification H3K4me3, an active epigenetic marker, on both frozen testicular sections and spread nuclei to assess MSCI at the pachytene stage. We observed a robust H3K4me3 signal across the entire chromatin and particularly on the XY body in *G6pc3* ^*‒*/ *‒*^ spermatocytes, whereas a very weak signal was detected on the entire chromatin of *G6pc3* ^+ / + ^ spermatocytes ( Figure 4C,E). The number of H3K4me3-positive signals on the XY body within single seminiferous tubules in wild-type mice was significantly greater than that in *G6pc3* ^*‒*/ *‒*^ mice ( Figure 4D,F). These findings suggest an active transcriptional status of the XY body in *G6PC3* ^*‒*/ *‒*^ spermatocytes. We subsequently performed RNA sequencing on wild-type and *G6pc3* ^*‒*/ *‒*^ testes. The results revealed significant upregulation of genes on the XY chromosome in the testicular tissue of *G6pc3* ^*‒*/ *‒*^ mice ( Figure 4G,H). GO functional analysis demonstrated that the upregulated genes are associated primarily with transcriptional activation ( Figure 4I). Hence, our findings indicate that G6PC3 is necessary for MSCI during spermatocyte development at the pachytene stage.

## Discussion

During premeiotic phase I of mouse spermatocytes, the pachytene phase typically spans approximately 6 days [ 14, 15]. This stage is characterized by crucial events such as crossover formation, XY body establishment, and MSCI, particularly in the middle stage of pachytene spermatocytes [ 16– 18]. Glucose 6 phosphatase-catalyzed 3 (G6PC3) was initially identified as a significant endogenous glucose-producing protein [19]. Despite several online databases indicating high specificity of *G6pc3* expression in male testicular tissue, its role in male germline function remains unexplored. Thus, our study represents the first investigation into the pivotal role of G6PC3 in spermatogenesis.

We revealed that G6PC3 is markedly overexpressed in mouse testicular tissue and plays a crucial role in the progression of pachynema during meiotic prophase I in mice. Notably, we observed a substantial signal intensity of G6PC3 in the XY body of spermatocytes. Through the generation of *G6pc3*-knockout mice, we established that *G6pc3* deficiency results in male azoospermia. Moreover, knockout of *G6pc3* halted the progression of pachynema in spermatocytes from the middle to late stages. In the absence of G6PC3, the XY body undergoes severe damage, as evidenced by the elongated, unaggregated morphology observed through H2AX staining. This abnormal XY tissue leads to failure of MSCI, subsequently resulting in spermatocyte arrest and apoptosis [20].

In mammalian spermatogenesis, transcriptional silencing of heterologous and largely unsynapsed sex chromosomes occurs via MSCI at the pachytene stage [21]. Proper formation of the XY body, a physically isolated compartment formed by a chromosomal protein, is crucial for MSCI [ 22, 23]. Studies have demonstrated that the absence of MSCI-related factors during meiosis results in the failure of XY body formation [ 8, 24, 25]. Our results revealed RNA Pol II and H3K4me3 signals on the sex chromosomes of the knockout spermatocytes, suggesting silencing failure. RNA-Seq data also revealed transcriptional activation in *G6pc3* ^‒/‒^ testes. This study presents the novel finding that G6PC3 is involved in the formation of functional XY bodies. Furthermore, failure of XY body formation leads to MSCI defects, which is detrimental to pachytene spermatocyte development.

In conclusion, our research highlights a novel role of G6PC3 in regulating meiotic silencing during mammalian spermatogenesis. Deficiency in *G6pc3* disrupts MSCI, resulting in complete meiotic arrest and cell elimination. This study reveals a new aspect of G6PC3 function in male meiosis, offering insights into whether mutations in *G6pc3* may contribute to nonobstructive azoospermia or related disorders.
